# Non-invasive Decoding of the Motoneurons: A Guided Source Separation Method Based on Convolution Kernel Compensation With Clustered Initial Points

**DOI:** 10.3389/fncom.2019.00014

**Published:** 2019-04-02

**Authors:** Mohammad Reza Mohebian, Hamid Reza Marateb, Saeed Karimimehr, Miquel Angel Mañanas, Jernej Kranjec, Ales Holobar

**Affiliations:** ^1^The Biomedical Engineering Department, Engineering Faculty, University of Isfahan, Isfahan, Iran; ^2^Brain Engineering Research Center, Institute for Research in Fundamental Sciences (IPM), Tehran, Iran; ^3^Department of Automatic Control, Biomedical Engineering Research Center, Universitat Politècnica de Catalunya BarcelonaTech, Barcelona, Spain; ^4^Biomedical Research Networking Center in Bioengineering, Biomaterials and Nanomedicine (CIBER-BBN), Barcelona, Spain; ^5^Faculty of Electrical Engineering and Computer Science, University of Maribor, Maribor, Slovenia

**Keywords:** electromyography, kalman filter, motor unit identification, neural decoding, source separation

## Abstract

Despite the progress in understanding of neural codes, the studies of the cortico-muscular coupling still largely rely on interferential electromyographic (EMG) signal or its rectification for the assessment of motor neuron pool behavior. This assessment is non-trivial and should be used with precaution. Direct analysis of neural codes by decomposing the EMG, also known as neural decoding, is an alternative to EMG amplitude estimation. In this study, we propose a fully-deterministic hybrid surface EMG (sEMG) decomposition approach that combines the advantages of both template-based and Blind Source Separation (BSS) decomposition approaches, a.k.a. guided source separation (GSS), to identify motor unit (MU) firing patterns. We use the single-pass density-based clustering algorithm to identify possible cluster representatives in different sEMG channels. These cluster representatives are then used as initial points of modified gradient Convolution Kernel Compensation (gCKC) algorithm. Afterwards, we use the Kalman filter to reduce the noise impact and increase convergence rate of MU filter identification by gCKC. Moreover, we designed an adaptive soft-thresholding method to identify MU firing times out of estimated MU spike trains. We tested the proposed algorithm on a set of synthetic sEMG signals with known MU firing patterns. A grid of 9 × 10 monopolar surface electrodes with 5-mm inter-electrode distances in both directions was simulated. Muscle excitation was set to 10, 30, and 50%. Colored Gaussian zero-mean noise with the signal-to-noise ratio (SNR) of 10, 20, and 30 dB, respectively, was added to 16 s long sEMG signals that were sampled at 4,096 Hz. Overall, 45 simulated signals were analyzed. Our decomposition approach was compared with gCKC algorithm. Overall, in our algorithm, the average numbers of identified MUs and Rate-of-Agreement (RoA) were 16.41 ± 4.18 MUs and 84.00 ± 0.06%, respectively, whereas the gCKC identified 12.10 ± 2.32 MUs with the average RoA of 90.78 ± 0.08%. Therefore, the proposed GSS method identified more MUs than the gCKC, with comparable performance. Its performance was dependent on the signal quality but not the signal complexity at different force levels. The proposed algorithm is a promising new offline tool in clinical neurophysiology.

## Introduction

A connectivity is generated between the motor-related areas of the brain during movement (Sporns et al., 2004; Rubino et al., 2006; Kim et al., 2017). A top-down structure in motor control is used particularly during an upper limb movement (with many degrees of freedom), where the brain and the muscles are functionally combined so that the muscle receives and electrically amplifies the resultant neural commands from the motor system (Haggard, 2008).

Over the last two decades, the non-invasive techniques for assessment of cortical and muscular activity have demonstrated significant progress and linear (e.g., coherence analysis) or non-linear (e.g., mutual information) methods have been used to analyze the relations between electromyographic (EMG), and electroencephalographic (EEG) signals (Chen et al., 2008; Meng et al., 2008). Due to the non-linear transfer functions of motor neurons, non-linear methods are more suitable for such a dependence analysis (Hashimoto et al., 2010; Ioannides and Mitsis, 2010) and for analysis of neural transfer functions between the central nervous system and pool of active motor units (MUs) (Negro and Farina, 2011; Gallego et al., 2015a,b).

Despite this progress in understanding of neural codes, the cortico-muscular coupling studies are still largely dependent on interferential EMG signal or its rectification for the assessment of motor neuron pool behavior (Gross et al., 2001; Schelter et al., 2009; Artoni et al., 2017). This assessment is not straightforward since the amplitude of surface EMG demonstrates several anatomical properties of the muscles, significantly interfering with the neural commands (Farina et al., 2010; Farina and Holobar, 2015, 2016). Indeed, amplitude or frequency of EMG are considerably affected by many factors, such as muscle anatomy, low-pass filtering of the subcutaneous tissues and MU Action Potential (MUAP) cancelation (Merletti and Farina, 2016). As result, they are not precisely related to the ongoing motoneuron activities and provide only a crude estimate of the central neural commands (so called neural drive) to the skeletal muscles (Farina et al., 2004, 2014a; Merletti and Farina, 2016). Therefore, the assessment of cortico-muscular connectivity and neuromuscular coupling via EEG–rectified EMG relationship is non-trivial and should be used with precaution.

Direct analysis of neural codes by decomposing the EMG (Webster et al., 2016), also known as neural decoding (Farina et al., 2014b; Karimimehr et al., 2017), is an alternative to EMG amplitude estimation (Gallego et al., 2017; Úbeda et al., 2017). It represents a paradigm shift, because it enables direct assessment of the neural drive to muscles (Farina and Holobar, 2015, 2016; Karimimehr et al., 2017). In fact, MU identification can be thought as a spike sorting algorithm (typical for computational neuroscience) applied to the outer layer of the human motor system (Balasubramanian and Obeid, 2011; Pani et al., 2016). The results of this MU identification can not only be used in EMG-EEG coupling analysis, but also in variety of research areas such as clinical neurophysiology for diagnosing neuromuscular disorders (Wheeler et al., 2006; Povalej BrŽan et al., 2017), sports and behavioral science (Merletti and Parker, 2004), movement science (Winter, 2009), robot-assisted rehabilitation (Savc et al., 2018), brain machine interface (Werner et al., 2016), and prosthesis control (Yoshida et al., 2010; Farina et al., 2014a).

In a typical experimental setup, EMG signals are detected using either conventional invasive intramuscular electrodes or non-invasive surface electrodes (Merletti and Parker, 2004). Both intramuscular (iEMG) and surface EMG (sEMG) signals include MUAP trains, superimposed into interferential signal patterns. Although iEMG provides some advantages, such as recording from deep muscles, it also has problems, such as discomfort and high selectivity. sEMG is thus a good alternative, particularly in sport sciences and studies of children (Ghaderi and Marateb, 2017). On the other hand, substantial MUAP superposition occurs in sEMG signals (Farina and Holobar, 2015, 2016). Also, surface MUAP shapes from different MUs are rather similar due to the volume conductor effects (Chen and Zhou, 2016). Thus, surface EMG decomposition is considered a very difficult task (Zhou and Rymer, 2004).

Variety of sEMG decomposition methods have been introduced in the literature. Generally, they either use shape-based algorithms, also called template matching (Xu et al., 2001; Gazzoni et al., 2004; Garcia et al., 2005; De Luca et al., 2006; Ren et al., 2006; Kleine et al., 2007; Nawab et al., 2008; Winslow et al., 2009; Siqueira Júnior and Soares, 2015) or the blind source separation (BSS) algorithm (Holobar and Zazula, 2004, 2007; Glaser et al., 2013; Ning et al., 2013, 2015; Chen and Zhou, 2016; Negro et al., 2016; Savc et al., 2018).

Although complementary fusion often results in more reliable findings (Durrant-Whyte, 1988), it is not yet sufficiently clear how to combine template-based and BSS sEMG decompositions, especially, as the aforementioned two methodological approaches differ substantially in the data model assumptions. For example, template-based algorithms assume a few recorded EMG channels and relatively low (or progressively increasing) muscle excitation levels in order to (progressively) identify the MUAP templates. They then use MUAP peel-off approach and combinatorial methods supported with artificial intelligence algorithms to identify each individual MU firing (Nawab et al., 2010). BSS approaches, on the other hand, model the mixing process of MUAPs in EMG, invert it and apply the inverse mixing procedure directly to the recorded EMG signals. For this reason, they do not rely on the initialization of MUAP templates, but do require relatively large number of recorded EMG channels to identify all the MU firings at once. In other words, BSS approaches estimate the MU filter directly in the space of MU spike trains and, once MU filter is estimated, apply it very efficiently (in terms of computational costs) to the recorded EMG signals, yielding all the MU firings at once (Holobar and Farina, 2014). Combination of these two fundamentally different sEMG decomposition approaches is non-trivial as it imposes complex methodological steps in which many parameters must be fine-tuned.

In this study, we focus on a hybrid sEMG decomposition approach that combines the advantages of both template-based and BSS decomposition approaches. Briefly, we use the single-pass density-based clustering algorithm to identify possible cluster representatives in different sEMG recording channels. Unlike traditional BSS-based decomposition algorithms, in which the initial search positions are randomly selected and checked on a trial-and-error basis, we use cluster representative samples as initial points. Similar approach was proposed in Ning et al. (2015), where *k*-means method was used to cluster MUAPs and, therefore, improve the initial estimate of MU filter in classical CKC approach. On the other hand, Chen et al. (2016, 2018a) and (Chen and Zhou, 2016) proposed the progressive FastICA framework along with the advanced peel-off and valley-seeking process that efficiently enhances the initialization of the MU filters and speeds-up the decomposition.

We then use the Kalman filter and a modified Gradient CKC (gCKC) algorithm (Holobar and Zazula, 2008) to further increase the rate of convergence of MU filter identification. Moreover, we introduce a new non-linear soft-thresholding algorithm to reduce the False Positives (FP) and False Negatives (FN) in MU spike post-processing. Thus, the proposed hybrid algorithm can be considered a “Guided Blind Source Separation” method.

The rest of the paper is organized as follows: in the next section, information about the signals and formulation of methods used in this study is presented. Section Results provides the results of the proposed method. The discussion is provided in section Discussion, along with conclusions.

## Materials and Methods

### Simulated Signals

A planar volume conductor model was used for generating synthetic sEMG signals (Farina and Merletti, 2001). Muscle, fat and skin tissues were used in the non-homogeneous and anisotropic volume conductor. It included an inimitable, and semi-infinite muscle layer with cross-section of 30 mm (transversal) × 15 mm (depth). The average fiber length, isotropic subcutaneous and skin layer thickness were 130 mm, 4 mm and 1 mm, respectively. Each MU had a random number of fibers uniformly distributed between 24 and 2,048 with the circular territories of 20 fibers/*mm*^2^. The conduction velocities were normally distributed (4.0 ± 0.3 m/s). In the initial recruitment, each MU discharged at 8 pulses per second (pps) (Fuglevand et al., 1993). Its discharge rate increased linearly with excitation (0.3 pps per % of muscle excitation). A grid of 9 × 10 (9 columns, 10 rows) monopolar surface electrodes with 5-mm inter-electrode distances in both directions was simulated. Fifteen sEMG signals with length of 16 s were generated and sampled at 4,096 Hz. Muscle excitation was set to 10, 30, and 50% Maximum Voluntary Contraction (MVC), yielding 262, 388, and 446 active motor units. Noteworthy, regardless the decomposition algorithm used, we can identify only superficial motor units, whereas small and distant motor units contribute to the physiological noise. In addition to these simulated physiological noise, colored Gaussian zero-mean noise with the signal-to-noise ratio (SNR) of 10, 20, and 30 dB and the bandwidth of 20–500 Hz was added to the raw surface EMG signals. Overall, 45 simulated signals were analyzed in this study. The simulated signals are available online: https://doi.org/10.6084/m9.figshare.5808291.

### The Proposed Algorithm

**Figure 1:**
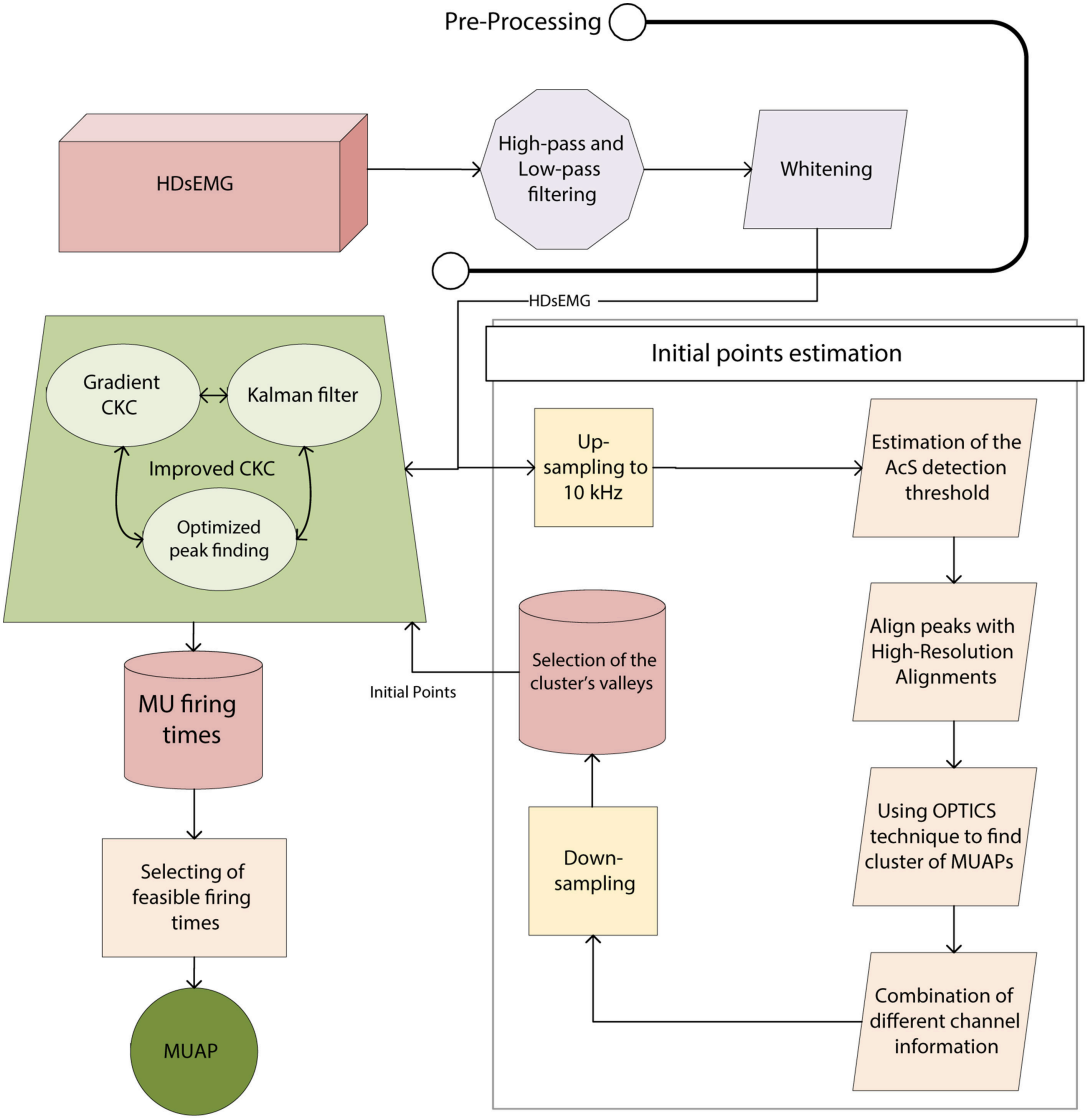
The schema of the proposed decomposition algorithm. The sEMG signal is first filtered using band-pass and whitening filters. The template-based clustering algorithm is then used to identify the initial points for the modified gCKC algorithm. Such a clustering algorithm includes the segmentation and high-resolution alignment of the up-sampled signal. Then, a modified density-based clustering OPTICS algorithm is used to automatically locate cluster representatives (i.e., templates) in different recording channels. Such information is combined for different channels and the peak samples of the decimated templates are used to initialize the modified g-CKC algorithm. Finally, Kalman filtering and optimized peak finding is implemented to increase the efficiency of the algorithm and also to reduce the decomposition errors.

The structure of the proposed algorithm is depicted in Figure 1. Briefly, the signal was passed to the first-order band-pass Butterworth filter with the cut-off frequencies of 20 and 500 Hz in the forward and reverse direction. Furthermore, a whitening method was performed on sEMG signals (Thomas et al., 2006). Then, the whitened signal was used for initial point estimation as well as for the modified gCKC. Moreover, the convergence rate of the gCKC algorithm was improved by the Kalman filter. The detailed information about each aforementioned step is provided in the sequel.

#### Whitening

Whitening, referred to as “convolutive sphering” (Thomas et al., 2006) was used as the first step of sEMG decomposition. Assume the following signal model,

(1) $$\begin{array}{l} \text{X}=\text{W}\text{Z} \end{array}$$

where **Z** is the *N* × *M* extended EMG signal matrix in which each row is a delayed repetition of one of EMG channels as proposed in Holobar and Zazula (2004) and Thomas et al. (2006) and each column corresponds to a time sample. In this study, the number of delayed repetitions of each signal is dependent to the sampling frequency and number of channels and in this work it was fixed to 30. The number of Whitening matrix **W** could be obtained, provided that the covariance matrix of **X** at time lag zero is equal to the identity matrix (Belouchrani et al., 1997):

(2) $$\begin{array}{l} W=\text{U}{\text{D}}^{-\frac{1}{2}}\;{\text{U}}^{T} \end{array}$$

where **D** is a diagonal matrix obtained by the eigenvalue decomposition of the covariance matrix of **Z** and **U** is the modal matrix:

(3) $$\begin{array}{l} E\left\{{\text{Z}\text{Z}}^{T}\right\}={\text{UDU}}^{T} \end{array}$$

with *E*{**ZZ**^*T*^} denoting the covariance matrix of **Z**.

#### Initial Point Estimation

Similar to the shape-based sEMG decomposition approach in De Luca et al. (2006), the time samples of the sEMG signals were up-sampled to 10 KHz to increase the time resolution (Figure 1). The resampling was based on band-limited approximation of the original signal, produced by inserting zeros into the discrete Fourier transform of the waveform (Crochiere, 1979). We also included steps from Stearns and Hush (2011) to reduce the end effects caused by the zero-insertion.

Next, the signal detection threshold, required by our segmentation, was calculated as follows: the absolute values of the EMG samples were calculated and sorted (vector ***s***). The square root values of cumulative sum of ***s*** were then calculated (***ss***). The maximum sample index *i*, where ***ss**(i)* multiplied by *K* exceeded the ***s**(i)* was found and ***s**(i)* was used as the detection threshold. In our study, *K* was empirically set to 4.0. The segmentation was then performed on each recording channel independently, using the estimated detection threshold on 2 ms intervals. Two-millisecond long signal segments, whose peak value was more than the detection threshold, were entitled as active segments (AcSs) (McGill et al., 2005).

Then, a high-resolution peak alignment method was used to align the detected AcSs on the highest peak (McGill and Dorfman, 1984). The time lags of the aligned AcSs were used as features for clustering. The density-based clustering was performed by using Ordering points to identify the clustering structure (OPTICS) algorithm (Ankerst et al., 1999; Daszykowski et al., 2002). This algorithm defines the clusters as areas of higher density. Among different density-based clustering algorithms, OPTICS was shown to be suitable for iEMG decomposition in which clusters have different dispersion (Marateb et al., 2011b). It is a single-pass clustering algorithm and unlike K-means or any other aggregative clustering method, does not require multiple runs on different predefined number of clusters (Daszykowski et al., 2002; Larose, 2005). The original OPTICS algorithm requires two parameters, namely the minimal number of points in a cluster MinPts and the Euclidean distance ε. A point *p* is called a “core point” if there are at least MinPts other points in its ε-neighborhood (*N*_ϵ_(p)). A point *p* is directly density-reachable from another point *q*, if *q* is a core point and *p* is in the ε-neighborhood of *q* i.e. *p*ϵ*N*_ϵ_(q). Point *p* is density-reachable from another point *q* if there is a chain of points *p*_1_*,…,p*_*n*_ where *p*_*i*+1_ is directly density reachable from *p*_*i*_ such that *p*_1_ = *q* and *p*_*n*_ = *p*.

By setting the parameter ε to the maximum distance between any points in the dataset, each point in the dataset will be the core point. Thus, the modified OPTICS algorithm only requires the parameter MinPts. In our case, MinPts was set to 40 for 10 s long signal epochs. OPTICS detects varying-density clusters (i.e., clusters with different dispersions) in the dataset, such that most similar points (AcSs) become neighbors. In the other words, similar points are grouped together into hierarchical clusters. In this procedure, the first AcS is selected as the current point. Its reachability distance (RD) is set to INF. Its nearest neighbor in respect to RD is then set as the current point. Its RD is calculated as the smallest density reachable distance from the previous AcS (Marateb et al., 2011b). This process is repeated until all the points are processed in this way. Using this method, the entire feature space is transferred to a two-dimensional plot in which x-axis corresponds to the ordered points and y-axis depicts the RD of the ordered points. Each RD valley in such plot is a possible cluster, with the point corresponding to the minimum RD in a valley as the corresponding cluster representative. In our study, the possible valleys were automatically extracted using the adaptive method proposed by Marateb et al. (2011b, 2012).

#### Modified CKC

Denote by **X**(*n, m*) the (*n,m*)-th element of the signal matrix **X** (the *m*-th sample of channel *n*) and by **X**(*m*) the m-th column of matrix **X**. The rows in matrix X are EMG channels and their delayed repetitions (Holobar and Zazula, 2004; Thomas et al., 2006). In the CKC algorithm (Holobar and Zazula, 2004, 2007), the spike train ${\overset{ˇ}{t}}_{j}$ of the *j-th* MU is estimated as

(4) $$\begin{array}{l} {\overset{ˇ}{t}}_{j}=R_{X{\overset{ˇ}{t}}_{j}}\prime R_{XX}^{-1}X \end{array}$$

where ${\text{R}}_{\text{X}\text{X}}^{-1}$ is the inverse of autocorrelation matrix of **X**, ${\text{R}}_{\text{X}{\overset{ˇ}{t}}_{j}}$ is the cross-correlation vector of **X** and ${\overset{ˇ}{t}}_{j}$ and the operator ′ denotes the matrix transpose. The cross-correlation vector ${\text{R}}_{\text{X}{\overset{ˇ}{t}}_{j}}$ is unknown, but can be iteratively estimated by gCKC (Holobar and Zazula, 2007, 2008):

(5) $$\begin{array}{l} {\text{R}}_{\text{X}{\overset{ˇ}{t}}_{j,g}}={\text{R}}_{\text{X}{\overset{ˇ}{t}}_{j,g-1}}+\eta \left(g\right){\left(f\left({\overset{ˇ}{t}}_{j,g}\right){\text{X}}^{\prime }\right)}^{\prime } \end{array}$$

where ${\overset{ˇ}{t}}_{j,g}$ is the *j*-th MU spike train, estimated in the *g-th* iteration, η is the step-size, *f* (*x*) = α(*x*) log (1 + *x*^2^) and α(*x*) is the attenuation coefficient introduced herein to improve the FP and FN errors in gCKC. The initial value of α is set to one but is then determined for each MU firing in each gCKC iteration. Namely, we observed that sometimes gCKC iterations erroneously amplify signal artifacts. Thus, after each iteration, we search for a spike detection threshold that yields the maximum Pulse-to-Noise Ratio (PNR) (Holobar et al., 2014).

(6) $$\begin{array}{l} PNR\left(j,k\right)=10\times log\left(\frac{\;E\left({\overset{ˇ}{t}}_{j,g}\right){|}_{{\overset{ˇ}{t}}_{j,g}\geq thr}}{\;E\left({\overset{ˇ}{t}}_{j,g}\right){|}_{{\overset{ˇ}{t}}_{j,g}<thr}}\right) \end{array}$$

where *thr* is the pulse detection threshold and PNR(*j,k*) is the Pulse-to-noise-ratio of the *j*-th MU in the k-th iteration. This is in fact a one-dimensional optimization problem and we efficiently solved it using a greedy search algorithm (Feo and Resende, 1995). The fitness function is the PNR while the optimization variable is the threshold *thr*. When this threshold is estimated, its 95% CI (Confidence Interval) is estimated and spikes within *thr* ± 95% CI of *thr* are detected (marginal spikes). The cluster representative (CR) of the spike samples surpassing the upper 95% CI threshold is formed and then if the corresponding sEMG AcS of a marginal spike has a high correlation with AcS of a CR, parameter α is set to one, otherwise it is set to 0.9. In this way, template-based methods are also used in the proposed modification of the previously introduced gCKC iterations (in addition to initial point estimation) (Holobar and Zazula, 2008). This operation is, in principle, equivalent to non-linear soft thresholding (Krzakala et al., 2016).

#### Kalman Filter

In our method, Equation (4) is substituted by expectation maximization Kalman filter (EM-Kalman filter), proposed by Bensaid et al. (2010). For this purpose, the gCKC is defined as the following state-space model joint to the EM-Kalman filter:

(7) $$\begin{array}{l} {\overset{ˇ}{x}}_{k|k-1}={\text{F}}_{k}x_{k-1|k-1}+w_{k}\\ =\left[\begin{array}{cc} {\text{I}}_{N} & \eta \left(g\right)\left(\text{X}f^{\prime }\left(t_{j,g,k-1}\right)\right)\\ 0 & 1 \end{array}\right]\left[\begin{array}{c} {\text{R}}_{\text{X}t_{j,g,k-1}}\\ 1 \end{array}\right]\\ +w_{k}\;{\overset{ˇ}{\text{t}}}_{j,g,k}\left(m\right)={\overset{ˇ}{\text{x}}}_{k|k-1}\text{H}\left(m\right) \end{array}$$

where ${\overset{ˇ}{\text{x}}}_{k|k\;}$and *k* are estimated state and current Kalman filter iteration, respectively, *g* is related to the *g*-th iteration of the gCKC algorithm and $w_{k}=C_{w}e_{k}=C_{w}[e_{k}(1)\;,e_{k}(2),\ldots ,e_{k}(N),0]\prime$ is the normally distributed noise vector of size (*N*+*1)* × *1* with variance $\sigma_{k}^{2}$. **C**_**w**_ is a diagonal *(N*+*1)* × *(N*+*1)* covariance matrix of noise and $e_{k}=V_{k}\prime {\overset{ˇ}{x}}_{k|k-1\;}$. According to the Bensaid et al. (2010), **V**_*k*_ is covariance matrix of ${\overset{ˇ}{\text{x}}}_{k|k-1\;}$ and due to the whitening process, **V**_*k*_ could be considered identity matrix. Thus, ${\text{w}}_{k}={\text{C}}_{\text{w}}{\overset{ˇ}{\text{x}}}_{k|k\;-\;1}$.

${\text{F}}_{k}=\left[\begin{array}{cc} {\text{I}}_{N} & \eta \left(g\right)\left(\text{X}f^{\prime }\left({\overset{ˇ}{t}}_{j,g,k}\right)\right)\;\\ 0 & 1 \end{array}\right]$ is the *(N*+*1)*×*(N*+*1)* state (or system) matrix with **I**_*N*_ denoting the identity matrix and *N* being the number of channels. ${\overset{ˇ}{\text{x}}}_{k|k\;}$ and **H***(m)* are defined by Equations (8) and (9):

(8) $$\begin{array}{l} \;{\overset{ˇ}{\text{x}}}_{k|k\;}=\left[\begin{array}{c} {\text{R}}_{\text{X}{\overset{ˇ}{t}}_{j,g,k}}\\ 1 \end{array}\right] \end{array}$$

(9) $$\begin{array}{l} \text{H}\left(m\right)=\left[\begin{array}{c} \text{X}\left(m\right)\\ 0 \end{array}\right] \end{array}$$

In this regard, a priori error covariance **P** and $\sigma_{k}^{2}$ could be defined by Equations (10) and (11), respectively.

(10) $$\begin{array}{l} P_{k|k\;-1}=F_{k}P_{k-1|k-1\;}F_{k}\prime \;+C_{w}C_{w}\prime \end{array}$$

(11) $$\begin{array}{l} \;\sigma_{k}^{2}=\frac{1}{M-1}\sum_{m=1}^{M}\left[H\prime (m)\left({\overset{ˇ}{x}}_{k|k\;}{\overset{ˇ}{x}}_{k|k}^{\prime }+P_{k|k\;}\right)H(m)-{\overset{ˇ}{t}}_{j,g,k}\left(m\right)\right] \end{array}$$

where **H** = [**H**(1), **H**(2), **H**(3)…**H**(*M*)] and its size is (*N* + 1) × *M*. Kalman gain (**K**) is defined with Equation (12).

(12) $$\begin{array}{l} K_{k}(m)=P_{k|k-1\;}H(m){\left(H\prime \left(m\right)P_{k|k-1\;}H\left(m\right)+\sigma_{k}^{2}\right)}^{-1} \end{array}$$

We then update gCKC algorithm and error covariance as:

(13) $$\begin{array}{l} {\overset{ˇ}{\text{x}}}_{k|k\;}\;={\overset{ˇ}{\text{x}}}_{k|k-1\;}\;+{\text{K}}_{k}\left({\overset{ˇ}{t}}_{j,g,k}-{\overset{ˇ}{t}}_{j,g,k-1}\right) \end{array}$$

(14) $$\begin{array}{l} \;P_{k|k\;}=P_{k|k-1\;}-K_{k}\;H\prime P_{k|k-1\;} \end{array}$$

When considering the state-space system matrix, *h*_*m*_ is the output matrix in the state-space model and **K**_*k*_ = [**K**_*k*_(1), **K**_*k*_(2), …, **K**_*k*_(*M*)] of size *(N*+*1)* × *M* is the Kalman gain in the *k*-th iteration. ${\overset{ˇ}{t}}_{j,g,k}$ in the first gCKC state is generated with the fixed point algorithm and one iteration of gCKC (Holobar and Zazula, 2007) and **P**_*k*−1|*k*−−1_ is initialized as **I***_N+1_* i.e., the identity matrix. The estimation of the observation noise power $\sigma_{k}^{2}\;$is achieved by maximizing the Log-Likelihood function $\log\left(P\left({\overset{ˇ}{t}}_{j}|\overset{ˇ}{\text{x}}\;,\sigma_{k}^{2}\right)\right)$ relative to $\sigma_{k}^{2}$ (Bensaid et al., 2010).

In a nutshell, we added additional noise factor in gCKC formulation and estimated it during each iteration since, practically, colored noise exists instead of white noise and gCKC algorithm, which works by correlation procedure, enhances colored noise (Li, 1990). This approach is used in other fields for finding noise parameters (Schwartz et al., 2015, 2016; Ge and Kerrigan, 2017) and also neuro spike decoding (Xue et al., 2017). In this light, EM-Kalman filter could prevent enhancing noise in ${\overset{ˇ}{\text{x}}}_{k|k\;}$ in each iteration of gCKC and decrease the FP errors. This hypothesis will be, at least partially, confirmed by the results presented in section Results as the newly presented method detects substantially more MUs than CKC method.

#### Motor Unit Identification

The non-linear soft thresholding method mentioned in section Modified CKC, was applied to the reconstructed MU spike trains to identify MU firing times. Moreover, extracted MUs were monitored in terms of regularity of the firing times and their distinguishability from the background noise. MUs with the PNR <20 dB were excluded from further validations (Holobar et al., 2014). The following firing time parameters were extracted and used for MU exclusion: the number of inconsistent firings times *n*_*I*_(number of pulses that are fired more than once in every 40 ms), the detection probability *p*_*d*_(the probability of MU inter-spike interval having normal distribution as assessed by the method proposed by McGill Kevin, 1984) and the mean discharge rate (MDR). A MU was excluded when *n*_*I*_ was higher than 50, or *p*_*d*_ was lower than 50% or MDR was higher than 35 Hz. The underlying assumptions were the following: during constant force isometric contractions, MU firing rates lie between 5 and 25 Hz. Moreover, the inter-spike intervals have a unimodal distribution which is approximately Gaussian (Clamann, 1969; Andreassen and Rosenfalck, 1980). The pseudocode of the proposed HDsEMG decomposition algorithm is available as the Supplementary Material.

### Performance Assessment

The identified MU firings were compared with the simulated firings. When at least 30% of the firings were time-locked to within ± 0.5 ms, a MUs was considered to be identified by our algorithm (Marateb et al., 2011a). For each identified MU, the accuracy of our new decomposition algorithm was assessed using the parameters of signal detection theory (e.g., TP (True Positive; correct firings), FN (False Negative; missed firings), and FP (False Positive; erroneous firings)). TP was the number of firings matching the simulated firings within ± 0.5 ms. FP was the number of firings not matching any simulated firing to within ± 0.5 ms. FN was the number of firings of the simulated MU that did not match any firings of the identified MU. Then, the performance indices Sensitivity (Se), Precision (Pr), and the Rate of agreement (RoA) were calculated (Holobar et al., 2010, 2014) (Table 1).

**Table 1:** Decomposition validation parameters.

**Parameter**	**Definition**
RoA	$\frac{\text{TP}}{\text{TP}+\text{FN}+\text{FP}}$
Sensitivity	$\frac{\text{TP}}{\text{TP}+\text{FN}}$
Precision	$\frac{\text{TP}}{\text{TP}+\text{FP}}$
DI_ki_	${\text{DI}}_{\text{ki}}\text{=}\frac{\text{min}\left\{\left\|{\text{m}}_{\text{ki}}\right\|,\left\|{\text{m}}_{\text{ki}}{-\text{m}}_{\text{k}*\text{i}}\right\|\right\}}{{\text{V}}_{\text{i}}^{\text{RMS}}}$
SIR(i)	$\left(1-\frac{\text{E}\left[{\left({\text{x}}_{\text{i}}\left(\text{n}\right)-\sum_{\text{j}}{\text{z}}_{\text{ij}}\left(\text{n}\right)\right)}^{2}\right]}{\text{E}\left[{\text{x}}_{\text{i}}^{2}\left(\text{n}\right)\right]}\right)\times 100$

RoA, Rate of agreement; SIR(i), signal-to-interference ratio of the i-th channel; DI_k,I_ is decomposability index and defines as the average MUAP shape of the k-th motor unit in the i-th channel, **m**_k*i_ is the MUAP most similar to **m**_**ki**_, among the other MUAPs in the same channel, ${\text{V}}_{\text{i}}^{\text{R}M\text{S}}$ is the RMS value of the channel i, and ||.|| stands for the Euclidean norm; TP (True Positive): the number of MU spikes correctly identified, FN (False negative): the number of missed MU spikes; FP (False Positive): the number incorrectly identified MU spikes; x_i_(n) denotes the time samples of the i-th surface EMG channel and **z**_**ij**_ stands for the time samples of the action potential train of the j-th motor unit reconstructed from the i-th EMG channel.

Moreover, the signal-to-interference ratio (SIR) was reported as the overall quality of the sEMG decomposition (Table 1). In fact, SIR estimates the percentage of the variance of the energy of the single-differential sEMG signals explained by the decomposition (Holobar et al., 2010). Also, the overall decomposability of a MU in the entire recording signals (cDI) was measured by the sum of norm of individual decomposability indices (DIs) (Holobar et al., 2010) (Table 1), calculated over all the EMG channels, normalized by the number of channels. The program was run on an Intel Core i7-8700 3.2 GHz CPU with 32 GB of RAM.

### Statistical Analysis

Continuous variables were reported as mean ± std. The level of statistical significance was set to *P* = 0.05. The normality of the variables was assessed using the Shapiro-Wilk test. We used generalized estimating equation (GEE) method (Hardin, 2005) to model factors associated with the repeated responses (RoA and the running time of the algorithm). The paired *t*-test was used to identify if there is a significant difference between the estimated MDR and CoV in the decomposed signals, compared with those of the simulated MU firings. The Pearson correlation coefficient (*r*) was used to assess the association between two normal variables. The statistical analysis was performed using SPSS version 16 (SPSS for Windows, Released 2007, Chicago, IL, USA, SPSS Inc.).

## Results

The average number of MUs, identified per contraction and SNR and also the accuracy of the decompositions of the assessed MUs are listed in Table 2. The accuracy of the clustering algorithm is also shown in this table as the average number of identified clusters. The cluster's representatives as well as their 20 nearby spikes in the RD plots, were compared with the simulated MU firings. When at least 75% of the firings in a cluster was time locked within 0.5 ms with firings of a simulated MU firings, the cluster was marked as correctly identified. Therefore, the reported number of identified clusters shows how many initial points were related to correct MU clusters. For comparison, the number of identified MUs and their decomposition accuracy of the gCKC (Holobar and Zazula, 2007) are also shown. Overall, our algorithm identified 16.41 ± 4.18 MUs with RoA of 84.00 ± 0.06%. The average number of modified gCKC iterations required to identify individual MU was 54.11 ± 3.06.

**Table 2:** Decomposition accuracy.

**SNR (dB)**	**MVC (%)**	**Average number of identified clusters**	**RoA**	**Sensitivity**	**Precision**	**Number of Identified MUs**	**SIR (%)**	**The Average Number of Iterations**	**Running time (sec) With the Kalman filter**	**Running time (sec) Without the Kalman filter**	**RoA (gCKC)**	**Sensitivity (gCKC)**	**Precision (gCKC)**	**Number of Identified MUs (gCKC)**
10	10	30	0.75 ± 0.17	0.80 ± 0.14	0.92 ± 0.09	11.60 ± 1.67	13.38 ± 3.76	51.2 ± 3.3	1739 ± 23 [704 ± 11]	1817 ± 7 [791 ± 5]	0.89 ± 0.12	0.95 ± 0.04	0.93 ± 0.09	9.6 **±** 1.5
10	30	28	0.76 ± 0.15	0.80 ± 0.13	0.93 ± 0.08	9.62 ± 1.81	14.04 ± 5.06	52.1 ± 2.1	1778 ± 20 [738 ± 6]	1862 ± 13 [811 ± 1]	0.89 ± 0.08	0.94 ± 0.04	0.93 ± 0.05	7.6 **±** 2.7
10	50	16	0.77 ± 0.16	0.81 ± 0.13	0.94 ± 0.08	11.00 ± 2.54	12.28 ± 5.49	49.3 ± 2.5	1846 ± 60 [740 ± 13]	1919 ± 22 [830 ± 10]	0.87 ± 0.09	0.93 ± 0.05	0.92 ± 0.06	7.3 **±** 1.5
20	10	27	0.89 ± 0.01	0.92 ± 0.02	0.96 ± 0.01	17.20 ± 2.62	15.05 ± 1.95	53.1 ± 3.7	1390 ± 14 [613 ± 2]	1647 ± 11 [680 ± 1]	0.94 ± 0.05	0.97 ± 0.02	0.96 ± 0.03	14.00 ± 1.87
20	30	28	0.88 ± 0.01	0.92 ± 0.01	0.95 ± 0.01	16.51 ± 2.20	15.11 ± 1.10	53.4 ± 3.3	1423 ± 15 [661 ± 23]	1687 ± 6 [704 ± 10]	0.91 ± 0.09	0.95 ± 0.03	0.95 ± 0.05	11.40 ± 3.58
20	50	20	0.88 ± 0.01	0.92 ± 0.01	0.95 ± 0.02	17.01 ± 2.71	15.42 ± 2.37	56.1 ± 2.5	1519 ± 11 [679 ± 18]	1721 ± 12 [718 ± 8]	0.90 ± 0.08	0.95 ± 0.04	0.94 ± 0.05	8.20 ± 1.92
30	10	28	0.90 ± 0.09	0.92 ± 0.07	0.98 ± 0.02	22.00 ± 3.01	18.12 ± 3.22	55.6 ± 1.3	1101 ± 29 [545 ± 47]	1539 ± 36 [670 ± 57]	0.94 ± 0.06	0.96 ± 0.03	0.96 ± 0.04	19.00 ± 0.71
30	30	29	0.90 ± 0.01	0.92 ± 0.02	0.98 ± 0.01	23.00 ± 1.10	17.39 ± 2.91	57.8 ± 2.1	1230 ± 28 [640 ± 18]	1652 ± 40 [703 ± 25]	0.92 ± 0.07	0.96 ± 0.04	0.94 ± 0.05	17.40 ± 2.51
30	50	21	0.90 ± 0.01	0.92 ± 0.01	0.98 ± 0.01	19.80 ± 1.00	15.01 ± 3.38	58.4 ± 2.4	1301 ± 28 [660 ± 27]	1822 ± 16 [718 ± 13]	0.91 ± 0.07	0.95 ± 0.04	0.94 ± 0.05	14.20 ± 3.11

*RoA, Rate of agreement; SIR: average signal-to-interference ratio. In gCKC, the number of iterations was fixed to 100. The total number of simulated MUs was 500. The program was run on an Intel Core i7-8700 3.2 GHz CPU with 32 GB of RAM. The reported running time was measured when analyzing 16 s of HDsEMG signals. For clarity reasons, the running time required to analyze 8 s long signals is also provided in squared brackets*.

The firing characteristics of the MUs, identified by the decomposition program and simulated MUs were very similar (Table 3), regardless the tested level of excitation and SNR.

**Table 3:** The firing statistics of the decomposed MUs.

**SNR (dB)**	**MVC (%)**	**MDR (Hz)**	**$\overset{ˇ}{\text{M}\text{D}\text{R}}\;$ (Hz)**	**CoV**	**$\overset{ˇ}{\text{C}\text{o}\text{V}}$**	**MDR bias (Hz)**	**CoV bias**	**cDI (%)**
10	10	13.17 ± 2.15	13.20 ± 2.19	0.14 ± 0.00	0.14 ± 0.00	0.03 ± 0.08	0.01 ± 0.00	15.40 ± 4.61
10	30	26.40 ± 7.27	26.74 ± 6.83	0.15 ± 0.00	0.14 ± 0.00	0.34 ± 0.24	0.01 ± 0.00	16.44 ± 8.37
10	50	30.19 ± 7.02	31.64 ± 6.02	0.15 ± 0.00	0.14 ± 0.00	1.45 ± 0.26	0.01 ± 0.00	18.19 ± 8.91
20	10	13.12 ± 2.10	13.20 ± 2.19	0.13 ± 0.02	0.14 ± 0.00	0.08 ± 0.08	0.01 ± 0.00	20.08 ± 4.44
20	30	26.77 ± 6.38	26.74 ± 6.83	0.13 ± 0.01	0.14 ± 0.00	0.03 ± 0.08	0.01 ± 0.00	19.87 ± 4.10
20	50	32.02 ± 4.26	31.64 ± 6.02	0.13 ± 0.02	0.14 ± 0.00	0.38 ± 0.14	0.01 ± 0.00	17.64 ± 4.53
30	10	13.18 ± 2.10	13.20 ± 2.19	0.13 ± 0.03	0.14 ± 0.00	0.02 ± 0.03	0.01 ± 0.00	22.63 ± 4.55
30	30	26.75 ± 6.33	26.74 ± 6.83	0.13 ± 0.04	0.14 ± 0.00	0.01 ± 0.01	0.01 ± 0.01	22.61 ± 4.80
30	50	31.88 ± 4.01	31.64 ± 6.02	0.13 ± 0.02	0.14 ± 0.00	0.24 ± 0.05	0.01 ± 0.00	21.89 ± 3.92

*MDR, Simulated mean discharge rates; $\overset{ˇ}{MDR}$: the MDR estimated by the proposed algorithm; CoV, Simulated coefficient of variability for the firing rate; $\overset{ˇ}{CoV}$, CoV estimated by the proposed algorithm; SIR: average signal-to-interference ratio; cDI: cumulative Decomposability Index. MDR and CoV biases were calculated as the absolute mean value of the pair-wise difference between estimated minus gold standard MDR and CoV, respectively*.

**Figure 2:**
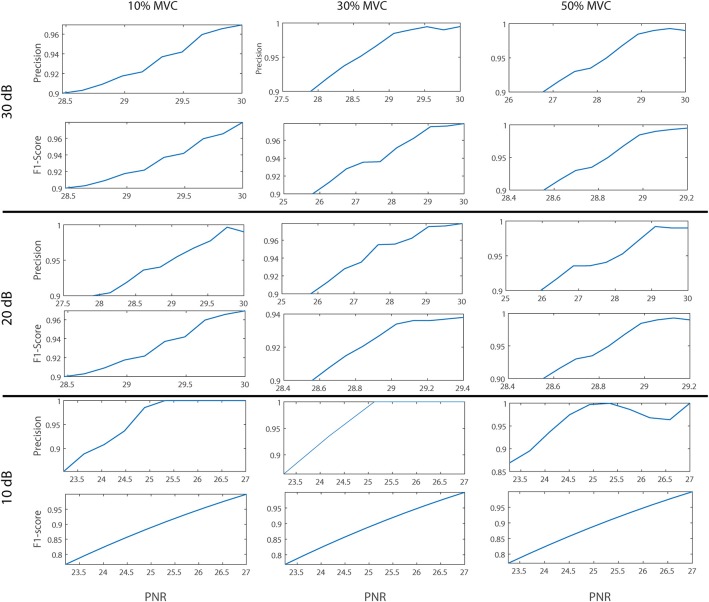
The sensitivity, precision, and Rate of Agreement (RoA) of the proposed algorithm vs. PNR (in dB). Representative plot is provided for each SNR level (10, 20, and 30 dB) at each simulated level of muscle excitation (10, 30, and 50% MVC).

**Figure 3:**
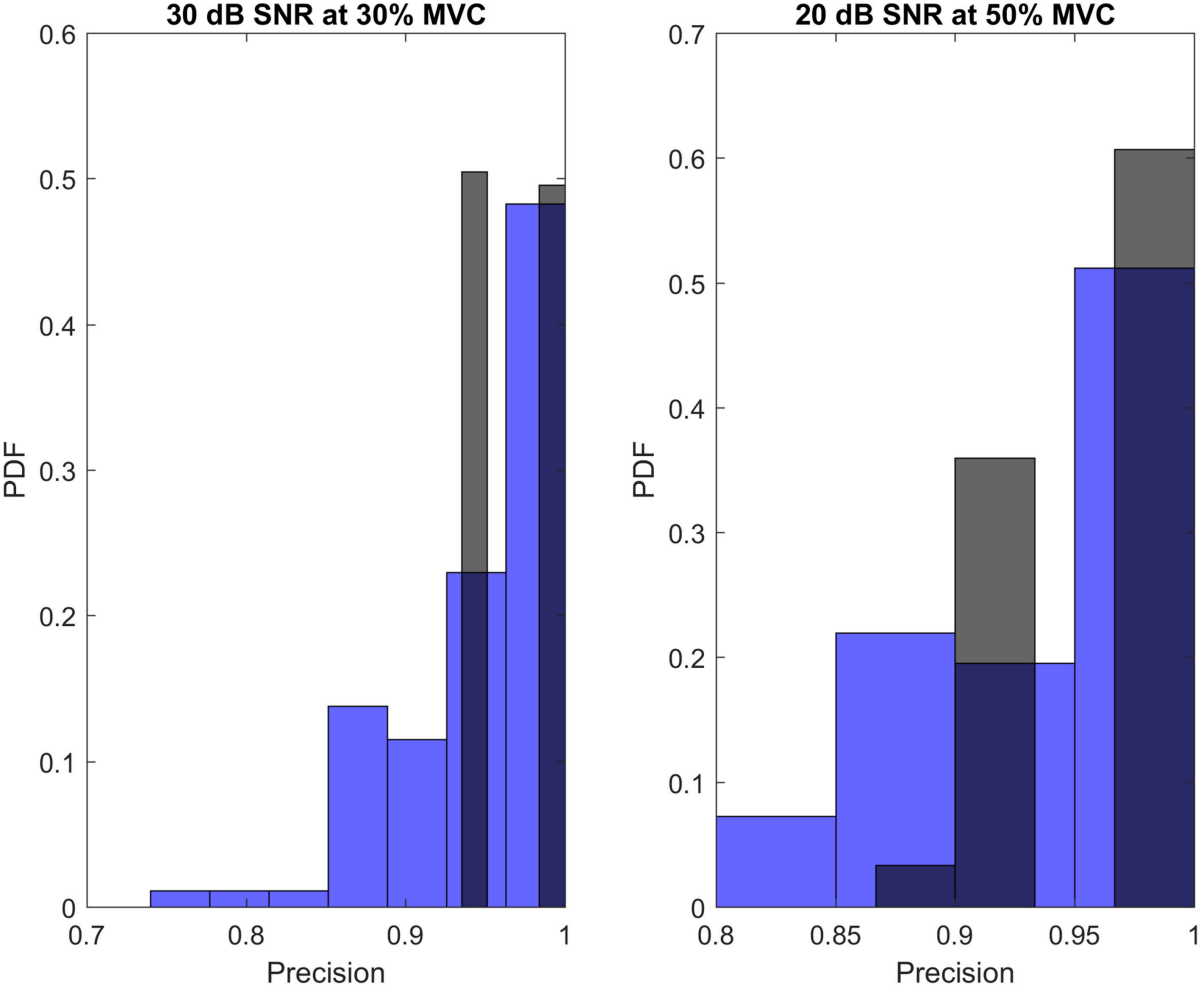
The histogram of the precision of the proposed decomposition algorithm (black), compared to the one form gCKC (blue) for 30 dB SNR at 30% excitation level (left), and 20 dB SNR at 50% excitation level (right).

The sensitivity, RoA and precision of the proposed algorithm vs. PNR for different excitation levels and SNR values are depicted in Figure 2. Also, the distribution of the precision of the proposed decomposition algorithm compared with that of the gCKC for 30 dB SNR at 30% excitation and 20 dB SNR at 50% excitation is shown in Figure 3.

**Figure 4:**
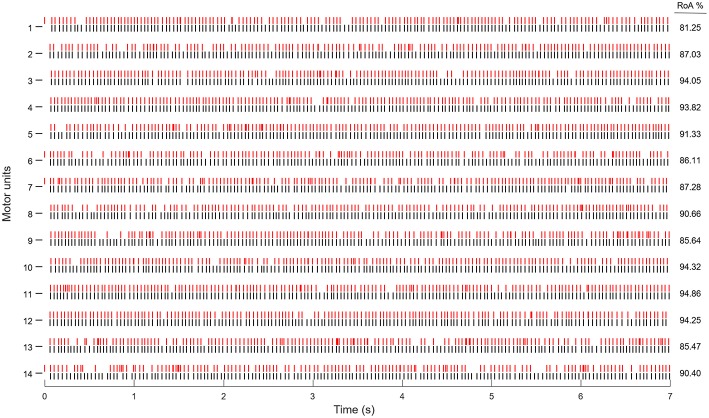
MU spike trains, identified from the simulated sEMG signal with 30 dB SNR and 10% excitation level (red), and the simulated firings (black). Each vertical line indicates one MU firing.

**Figure 5:**
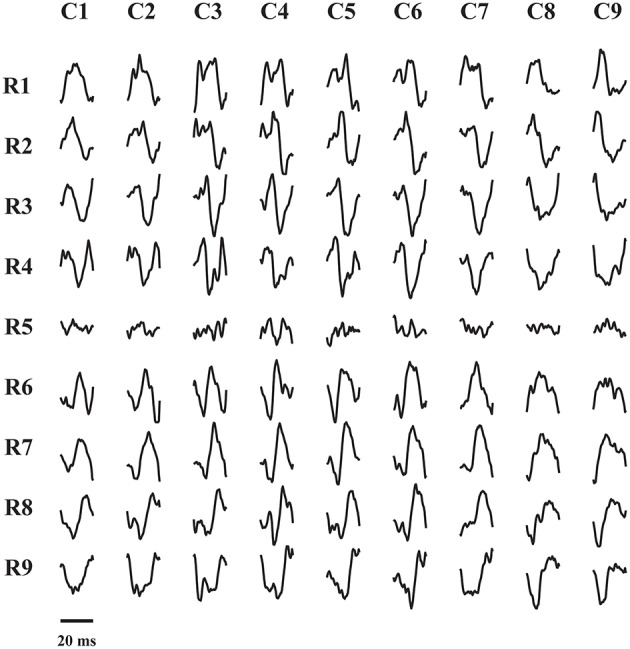
Single-differential sEMG MUAP waveforms of MUs from different sEMG channels. The corresponding MU was identified with accuracy >90%.

Figure 4 shows an example of the MUAP trains identified during seven seconds of a contraction at 10% MVC and 30 dB SNR. In this case, 14 MUs were identified with the average RoA of 89.00 ± 0.89 (%). Figure 5 shows the spatial distribution of Single-differential sEMG MUAP of MU 14 form Figure 4 as estimated by the spike-trigger averaging of the HDsEMG signals over the estimated MU firing times.

## Discussion

Neural decoding is used to identify how the electrical activity of neurons generates responses in the brain (Jacobs et al., 2009). In the case of motor system, this can be performed either at the level of motor nerves or motor units. Although, the methods are very similar, the former is called “spike sorting” in computational neuroscience while the latter is known as “EMG decomposition,” or “decoding of the neural drive to the muscles” (Rey et al., 2015; Webster et al., 2016; Karimimehr et al., 2017). Such a decomposition algorithm could be used in variety of applications, including prosthesis control (Farina et al., 2014a) or robot-assisted neurorehabilitation (Savc et al., 2018). The structure of the spike sorting algorithms is, in principle, similar to that of iEMG decomposition methods, where the recording electrodes are close to the MU fibers. However, this is not the case for sEMG decomposition, where electrodes are located over the skin at a relatively large distance from the muscle fibers.

sEMG decomposition is considered a very difficult task when low selectivity of traditional sEMG electrodes, low-pass filtering of MUAPs, their overlapping, and shape similarities are considered (Farina et al., 2004). In this study, we proposed a new algorithm that combines the advantages of two different approaches to sEMG decomposition, namely the template-based and the BSS algorithms. Classification procedures are critical steps of the template-based algorithms. sEMG MUAPs are rather similar in shape and their classification is challenging (De Luca et al., 2006; Nawab et al., 2010). BSS algorithms, on the other hand, combines MU activities in the space of MU spikes in order to identify all the MU firings (Holobar and Farina, 2014). Many BSS algorithms cannot guarantee the identification of the same set of sources in multiple runs, because they rely (at least partially) on stochastic algorithms, for example on random initialization of vector ${\text{R}}_{\text{X}{\overset{ˇ}{t}}_{j}}$in Equation (5).

Our algorithm, is fully deterministic since it identifies the initial points using a new clustering algorithm (Figure 1) rather than random initialization. Other CKC-based algorithms, use the time samples of the peaks of the sEMG signal as the possible starting point of the gradient-based optimization. However, sEMG contains highly overlapped MUAPs, and selected peaks of the signal usually correspond to the overlapped MU activity. This overlapped MU activity is then separated in several iterations of gCKC algorithm (Equation 5). On the other hand, if the initial ${\text{R}}_{\text{X}{\overset{ˇ}{t}}_{j}}$ estimate is not related to any MU firings, but rather to the movement artifact or line interference, the CKC algorithm cannot amplify the MU spikes and the noise is amplified instead. Thus, being fully deterministic in our sense means that we know where a MU firing exists and we start the CKC gradient optimization from the initial point with a much higher MU identification potential than gCKC algorithm.

Similar aggregative clustering methods have been proposed in the past for sEMG decomposition (Ning et al., 2015). However, aggregative clustering methods, such as K-means or Fuzzy C-means do not have satisfactory “rerun” stability due to poor reproducibility based on random initialization (Jayaram and Klawonn, 2013). Furthermore, they may get stuck in local minimum owing to a two-stage iterative algorithm used to minimize the sum of point-to-center distances over all clusters. Finally, they have high computational complexity due to the need of running the algorithm several times with the pre-defined number of clusters (Duda et al., 2001). Therefore, we used a density-based OPTICS algorithm, which is a single-pass algorithm designed particularly for high-dimensional data (Ankerst et al., 1999; Daszykowski et al., 2002). This algorithm can automatically identify the optimal number of clusters when a proper automatic valley detection algorithm is used (Marateb et al., 2011b). Moreover, it adaptively identifies clusters of different sizes and variable dispersions and densities (Loh and Park, 2014).

In sEMG, there are many active MUs. This causes the overlap between MUAPs and thus the number of isolated segments reduces significantly, especially at higher contraction levels. Although each recording sEMG channel provides a different perspective of the MUAPs, the performance of the initial point detection generally drops at higher contraction levels (Table 2; The number of identified clusters variable). This is one of the limitations of the proposed algorithm. Using different spatial filters could improve the performance of this step of the algorithm which will be investigated in our future work.

Moreover, several cluster representatives identified by the OPTICS algorithm may be related to the same MU. This is why the average number of clusters identified by the OPTICS algorithm is higher than the number of identified MUs by the entire algorithm (Table 2). Since the MUAP shapes are very similar over the skin and largely submerged into the physiological noise, it is not possible to merge them unless their firing times are identified by the CKC gradient loops. On the other hand, due to the high overlapping between different MUAPs and the similarity between the shapes of different MUAPs in the sEMG signal, it is not optimal to use traditional classifiers (e.g., the minimum-distance classifier) based on the clusters obtained by the OPTICS algorithm. Thus, the combination of the OPTICS and gCKC algorithm is required, as proposed in our manuscript.

In our study, results of clustering were used as initial points in a modified g-CKC algorithm (Figure 1). In fact, our algorithm is a Guided-Source Separation (GSS) method with the following modifications introduced into the gCKC method:

1. An adaptive soft-thresholding method was designed based on stochastic optimization, correlation analysis and estimation theory on the spike times as to reduce the FP (by suppressing noise spikes) and FN (by amplifying valid marginal spikes) error rates;
2. The Kalman filtering was used to improve the convergence rate of the algorithm and to reduce the noise enhancement in gCKC iterations.

The performance of our algorithm was compared with that of the previously published gCKC algorithm (Table 3) since gCKC was shown to be accurate with different muscle architectures (Holobar et al., 2010; Marateb et al., 2011a; Webster et al., 2016) and different pathological conditions including essential tremor (Gallego et al., 2015a), Parkinson's disease (Holobar et al., 2012), diabetes (Watanabe et al., 2013), after stroke (Li et al., 2015), targeted muscle reinnervation (Farina et al., 2014c), and cleft lip surgery (Radeke et al., 2014). The previously introduced PNR, an efficient objective signal-based metric of gCKC decomposition accuracy (Holobar et al., 2014), was also reported for our decomposition results (Table 2, Figure 2). Although the sensitivity and RoA of the proposed algorithm is less than that of the gCKC algorithm, we identified more MUs at comparable Precision (Table 2; The average precision of 0.96 ± 0.05 compared with 0.95 ± 0.05 for gCKC). In fact, as mentioned in section Kalman filter, the Kalman filter decreases the FP errors and such errors have a direct effect on the Precision (Table 1). Therefore, FP error reduction goal was achieved by Kalman filter and the adaptive soft thresholding method. In our future work, we intend to focus on the reduction of FN errors. Moreover, we tested our algorithm with and without the Kalman filter. With the Kalman filter, the number of iterations was reduced in average down to 48% compared to the case without the Kalman filter. Moreover, when the Kalman filter was added, two more MUs were correctly identified, on average, whereas the running time of our algorithm was significantly reduced (*P* < 0.001; GEE).

The results of our statistical analysis showed that RoA was significantly associated with the SNR parameter i.e., the signal quality (*P* < 0.001; GEE) but not with the muscle excitation level i.e., signal complexity (*P* > 0.05; GEE). RoA was significantly and highly correlated with cDI at 10 dB and 20 dB SNR (*P* < 0.001; *r* > 0.7), but not at 30 dB SNR. This suggests that the proposed algorithm would work even if MUAPs were relatively similar in shapes when the quality of the signal was high. Moreover, there was no significant differences between the MDR and CoV values estimated from the decomposed sEMG signals and the corresponding simulated MU firings (*P* > 0.05; paired *t*-test). Thus, the decoded neural information can be considered reliable. Although measuring the MU identification accuracy indirectly, the PNR parameter was highly correlated with all the performance indices, namely with RoA, Sensitivity, and Precision (Figure 2; *P* < 0.001, *r* > 0.8). Thus, MUs with high PNR values can be considered as accurately identified. This is in agreement with the results in Holobar et al. (2014); Martinez-Valdes et al. (2016), and Watanabe et al. (2016).

In our study, simulated data was used for validation. Although, the used volume conductor model has been widely discussed in the literature (e.g., in McGill, 2004; Zazula and Holobar, 2005; Holobar et al., 2009; Zalewska, 2009), it could not completely resemble the experimental sEMG signals (De Luca and Nawab, 2011). Thus, further tests on experimental signals from different skeletal muscles in different experimental conditions (i.e., different contraction levels, force profiles, muscle geometries etc.) are required before the reported results can be generalized. This is a common challenge to all the reported HDsEMG decomposition algorithms as the results from one experimental setup cannot easily be generalized to all experimental conditions. In this study, we introduced the novel methodological steps and tested their efficiency against previously introduced CKC method that has been extensively validated over the past decade and applied to many (but not all) experimental conditions. Further generalization of the results reported herein exceeds the scope of this study and is left for the future work.

Indeed, the validation of the decomposition on experimental sEMG signals is, in principle, controversial. Using cross-checking, also known as two-source method, between the decomposition of concurrently recorded iEMG and sEMG signals, could only assess the accuracy of common MUs. Partitioning the sEMG channels into two groups and cross-checking them against each other, on the other hand, is also problematic as the information they convey is highly correlated (Webster et al., 2016). However, the objective measure PNR could be reported and used as the MU reliability index by the decomposition program (Holobar et al., 2014). Moreover, a novel validation approach proposed by Chen et al. could serve as a supplement to the conventional two-source methods (Chen et al., 2018b).

Finally, the proposed GSS algorithm has relatively low sensitivity to MUs, although it identifies more MUs than gCKC (Table 2). It identifies only a small portion of active MUs. This is a common limitation to all EMG decomposition approached introduced by now. The proposed algorithm is also limited to off-line analysis and cannot easily be converted to the online neural decoding algorithms (Glaser et al., 2007; Karimimehr et al., 2017). Its online implementation requires improvements in the rate of convergence of gradient-based optimization (Equation 5). For this purpose, the condition number of the Hessian matrix could be analyzed and proper diagonal scaling could be used to reduce the number of iterations (Beck, 2014), but this additional step has not been thoroughly investigated yet. Moreover, detection of firing-time inconsistency could be added to the OPTICS clustering algorithm in order to increase the number of MUs identified by the entire algorithm. We will address these methodological improvements in our future work.

In conclusion, we proposed a new framework for sEMG decomposition. The proposed algorithm is promising and a new offline tool in clinical neurophysiology.

## Author Contributions

MRM, HM, SK, and AH participated in conceptualization, data curation, and validation. MRM, HM, SK, MAM, JK, and AH participated in formal analysis, investigation, and methodology. HM, MAM, and AH participated in project administration and resources. MRM, HM participated in software, and visualization. HM and AH supervised the project. MAM and AH acquired funding. MRM, HM, JK, and AH contributed to writing the original draft and SK, MAM revised the manuscript. All authors read and approved the final version of the manuscript and agreed for all aspects of the work.

### Conflict of Interest Statement

The authors declare that the research was conducted in the absence of any commercial or financial relationships that could be construed as a potential conflict of interest. The reviewer PZ declared a past co-authorship with one of the authors, AH, to the handling Editor.
