# Supplementary material for: Non-invasive Decoding of the Motoneurons: A Guided Source Separation Method Based on Convolution Kernel Compensation With Clustered Initial Points
Source: Front Comput Neurosci. 2019 Apr 2;13:14. doi: 10.3389/fncom.2019.00014 (PMC6455215; doi:10.3389/fncom.2019.00014)
Supplement: Supplementary file 1 [file Table_1.DOCX]

Supplementary Material

The pseudo code of the HDsEMG decomposition algorithm:

# comments:

# X is a n*m matrix, which n is number of channel and m is number of samples

# T is spkie firing Time

**Funtion** Proposed_Method:

1. Pre-processing Function

2. Initial_Point_Estimation Function

3. For each selected initial point do:

3.1. Name X_m as m-th column of matrix X in which m is initial point

3.2. Use Equation 8 (fixed point algorithm) by replacing initial

point with cross-correlation of X and T to find the firing time vector

3.3. Create attenuation coefficient and set it to one for all time

3.4. While Termination Criteria is not met do this:

3.4.1. Use Equation 9 to estimate new cross-correlation of X and T

3.4.2. Use greedy search for finding optimize threshold for maximizing PNR

3.4.3. set attenuation coefficient based on detected threshold

3.4.4. Initialize P, F, G, h_m and state

3.4.5. Estimate variance of noise

3.4.6. Find Kalman Gain and then update P and state

3.4.7. Find T with state and output of state-space model

EndWhile

EndFor

**Endfunction**

**Function** Pre-processing:

Pass In X

1. Apply the spatial filter

2. Apply traditional band-pass filter

3. Whitening Function

Pass Out Whitened X

**Endfunction**

**Function** Whitening:

Pass In X in which rows are channels of sEMG

1. Detrending channels seperately respecting to their averages

2. Normalize each channel with linear normalization

3. Add K number of lag for each channel and insert them as a row of X

4. Calculate the covariance matrix of X

5. Use eigenvalue decomposition for finding modal matrix U and

diagonal matrix D including eigen values

6. Calculate W

Pass Out W*X

**Endfunction**

**Function** Initial_Point_Estimation:

Pass In X

1. For each channel do:

1.1 Up sampling channel to 10 kHz

1.2 Estimate the thresold and length of spikes

1.3 Extract spikes

1.4 Using High-resoloution alignment for aligning peaks

1.5 Using OPTICS clusterring algorithm to cluster spikes

1.6 Down sampling channel to previous frequency

Pass Out Centers of Clusters

EndFor

2. Combine information of Clusters for all channels

3. Pass Out centers of all cluster for all channles

**Endfunction**
